# Effect of blockage ratio on flow of a viscoelastic wormlike micellar solution past a cylinder in a microchannel†

**DOI:** 10.1039/d2sm01162j

**Published:** 2022-11-14

**Authors:** Cameron C. Hopkins, Amy Q. Shen, Simon J. Haward

**Affiliations:** Okinawa Institute of Science and Technology Graduate Univerisity Onna-son Okinawa 904-0495 Japan c.c.hopkins91@gmail.com simon.haward@oist.jp

## Abstract

We present experiments on the flow of a viscoelastic wormlike micellar solution around cylinders (radius *R*) confined in straight microchannels (width *W*). Thirteen flow geometries are tested where the blockage ratio is varied over a wide range 0.055 ≤ *B*_R_ = 2*R*/*W* ≤ 0.63. Experiments are performed at negligible Reynolds number, and for Weissenberg numbers *Wi* = *λU*/*R* up to 1000, where *U* is the average flow speed and *λ* is the relaxation time of the fluid. Micro-particle image velocimetry is used to characterise the flow state at each *B*_R_ and *Wi*. In all of the geometries, a first critical Weissenberg number marks a transition from symmetric flow to an asymmetric but time-steady flow state, while a second higher critical Weissenberg number marks the onset of time-dependent flows. However, we report a clear shift in behaviour over a narrow intermediate range of 0.33 ≲ *B*_R_ ≲ 0.41. Channels with *B*_R_ ≤ 0.33 fall in a ‘low’ *B*_R_ regime, with instabilities that originate from the downstream stagnation point, while those with *B*_R_ ≥ 0.44 fall in a ‘high’ BR regime, with instabilities developing at the upstream stagnation point. Behaviour within the newly-identified intermediate *B*_R_ regime is complex due to the competing influence of the two stagnation points. We summarise all our results in a flow state diagram covering *Wi*–*B*_R_ parameter space, clearly defining the different regimes of blockage ratio for the first time. Our results contribute to the understanding of the complexities of viscoelastic flow in this benchmark geometry.

## Abstract 2

We present experiments on the flow of a viscoelastic fluid past a cylinder in a microchannel. Different elastic flow instabilities are examined and summarised in a flow state diagram covering Weissenberg number – blockage ratio parameter space.

## Graphical abstract

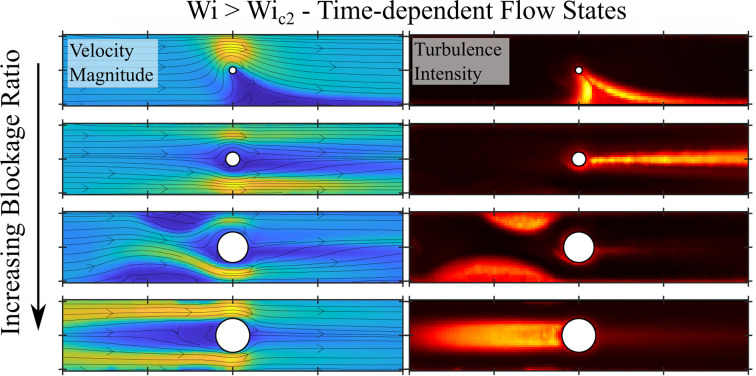

## 1 Introduction

**Fig. 1:**
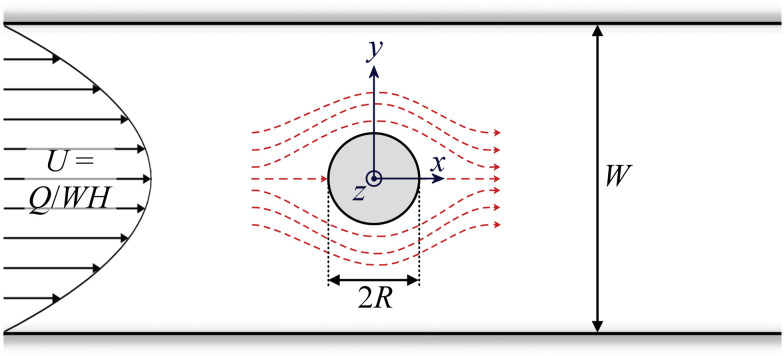
Schematic drawing of the flow geometry with a circular cylinder of diameter 2*R* located in the centre of a channel of width *W*. The extent to which the cylinder blocks the channel (*i.e.*, the ‘blockage ratio’) is *B*_R_ = 2*R*/*W*. The coordinate system originates from the centre of the cylinder and the height of the channel through *z*-direction is denoted *H*. The average velocity of the Poiseuille-like flow in the channel upstream of the cylinder is *U* = *Q*/*WH*, where *Q* is the volumetric flow rate imposed by a syringe pump. Dashed red lines with arrows indicate streamlines in the vicinity of the cylinder expected for a Newtonian creeping flow.

Developing an accurate prediction of the viscoelastic flow around a cylinder in a channel is a fundamental problem in non-Newtonian fluid mechanics. The specific case of a cylinder of radius *R* located in the centre of a straight channel of width *W* = 4*R*, such that the ‘blockage ratio’ *B*_R_ = 2*R*/*W* = 0.5 (Fig. 1), has been considered as one of the ‘benchmark’ non-Newtonian flows for testing the predictions of constitutive equations against experimental results.^1^ However, it has long been recognised that the choice of *B*_R_ = 0.5 is somewhat arbitrary, and that varying *B*_R_ around this value can result in substantially different behaviour of the fluid.^2,3^

Flow past a cylinder confined in a channel (Fig. 1) presents a complex mix of shearing and extensional flow kinematics. Shear rates increase and then decrease as fluid squeezes through the gaps between the cylinder and the channel walls. This squeezing also amounts to a transient extensional (contraction/expansion) flow. In addition, axial stagnation points exist at the leading and trailing edges of the cylinder, which result in shear-free and persistent extensional kinematics with high residence times.

For microstructured viscoelastic fluids such as polymeric or wormlike micellar solutions, with a rheology that depends on the imposed shear and/or extensional deformation rate, the combination of kinematics encountered in flow past a cylinder can lead to unexpected and complex dynamical behaviour and instabilities. Comprehensive recent reviews on the topic are available in ref. 4 and 5. We focus here only on microfluidic experiments on flows around cylinders that conveniently allow the effects of inertia on the flow dynamics to be neglected due to the small characteristic length scales involved.^6^ In this case, at low average flow velocities around the cylinder, *U*, a viscoelastic fluid will largely behave in a ‘Newtonian-like’ way, exhibiting streamlines that are symmetric with respect to both the *x* and *y*-axes (as indicated in Fig. 1). However, as *U* is increased beyond a critical value, non-Newtonian effects may be observed. For higher blockage ratios (*B*_R_ ≳ 0.5), the most dominant effects are manifested upstream of the cylinder, for example with the occurrence of large recirculating regions fixed at the upstream stagnation point.^7–10^ However, at lower blockage ratios the deviation from Newtonian-like behaviour is most obvious downstream of the cylinder, for example with regions of low flow velocity forming a trailing wake that may extend for many cylinder radii.^11–15^ In both cases, the critical flow velocity for the onset of non-Newtonian behaviour typically scales with the dimensionless Weissenberg number *Wi* = *λγ̇*. Here, *λ* represents a characteristic relaxation time of the fluid, and *γ̇* represents a characteristic deformation rate (shear or extensional) in the flow field. For *Wi* ≳ 0.5, the microstructure can be deformed by the flow, giving rise to rheological effects such as shear thinning in regions of shearing kinematics and extension thickening (or elasticity) in regions of extensional kinematics.^16^ Varying *B*_R_ modifies the relative importance of the various kinematic features in the flow field around the cylinder, and thus modifies the way in which a given viscoelastic fluid will respond for *Wi* ≳ 0.5.

Other non-Newtonian flow features may also be observed depending on the fluid rheology, the blockage ratio and/or the magnitude of the imposed Weissenberg number. For instance, the buckling of streamlines upstream of the cylinder has been observed in fluids of various rheology and *B*_R_.^12,17,18^ However, the development of strong lateral flow asymmetry around, and downstream of, the cylinder has so far been exclusively reported at low *B*_R_ ≤ 0.1 and with fluids exhibiting both strong shear thinning and elasticity.^13–15,19^ Recently, with a shear thinning and elastic wormlike micellar solution, the occurrence of wall-attached vortices upstream of a cylinder at *B*_R_ = 0.5 has also been reported.^18^

In the present work, we employ a single viscoelastic fluid (a shear thinning and elastic wormlike micellar solution) with model Maxwellian relaxation dynamics, and we study its flow behaviour for Weissenberg numbers up to 1000 around cylinders presenting a wide range of blockage ratios 0.055 ≤ *B*_R_ ≤ 0.63. Such a wide ranges of *B*_R_ and *Wi* in combination have not previously been examined for viscoelastic flows around cylinders. A total of thirteen different blockage ratio cylinders are contained in microchannels of high aspect ratio (*A*_R_ = *H*/*W* = 5), such that the flow can be assumed to be inertialess and two-dimensional (2D), greatly facilitating the possibility of comparison with numerical modelling. In each channel, several distinct flow states are observed as *Wi* is increased. The large number of channels with incrementally varying blockage also allows trends of behaviour to be followed with *B*_R_. A flow state diagram in *Wi*–*B*_R_ parameter space, reveals an abrupt transition in behaviour for 0.33 ≲ *B*_R_ ≲ 0.41, defining a clear boundary between the ‘low’ and ‘high’ *B*_R_ regimes. This ‘phase diagram’ will be a valuable aid for predicting the nature of viscoelastic flows around cylinders as a function of fluid rheology, imposed flow rate, and channel geometry. Furthermore, it provides a comprehensive benchmarking target for reproduction in future numerical simulations.

## 2 Experimental methods

### 2.1 Test fluid rheology

The test fluid is a strongly shear thinning (in fact, shear banding)^20^ and viscoelastic semidilute and entangled aqueous wormlike micellar solution composed of 100 mM cetylpyridinium chloride and 60 mM sodium salicylate (100 : 60 CPyCl : NaSal).^21,22^ The fluid is identical to that used in a recent study of the flow past a microfluidic cylinder of *B*_R_ = 0.5,^18^ and similar to that used in an earlier related study in which *B*_R_ = 0.1.^13^ At the ambient laboratory temperature (24 °C), the fluid has a zero shear viscosity *η*_0_ ≈ 27.5 Pa s. With increasing shear rate *γ̇*, the viscosity *η*(*γ̇*) thins dramatically with a power law index *n* ≈ 0 towards a high-shear-rate plateau value of *η*_∞_ = 2 mPa s. The linear viscoelastic response of the fluid is well-described by a single mode Maxwell model that provides a terminal relaxation time of *λ* = 1.54 s. Full details of the rheological characterization of the fluid, also including measurements of the first normal stress difference, are available in ref. 18.

### 2.2 Microchannel design and experimental setup

Microchannels are fabricated in fused silica glass by the technique of selective laser-induced etching using a LightFab instrument (LightFab GmbH).^23–25^ Each channel has height *H* = 2000 μm and width *W* = 400 μm, and thus aspect ratio *A*_R_ = *H*/*W* = 5 (see Fig. 1). Cylinders have radii *R* = 11, 20, 30, 40, 58, 66, 76, 80, 88, 96, 100, 116, and 126 μm, thus providing respective blockage ratios of *B*_R_ = 2*R*/*W* = 0.055, 0.1, 0.15, 0.2, 0.29, 0.33, 0.38, 0.4, 0.44, 0.48, 0.5, 0.58, and 0.63. Flow through the microchannels is imposed using two Nemesys low pressure syringe pumps, one of which infuses fluid at the upstream inlet to the channel, and the other of which withdraws fluid from the downstream outlet (both at the same volumetric flow rate *Q*). Each channel is configured with a distance of 12 500 μm (= 31.25*W*) between the inlet and the cylinder, in order to allow the flow to become fully developed upstream of the cylinder, and an equal distance between the cylinder and the outlet to allow redevelopment of the flow in the downstream.

The average flow velocity in the channel is *U* = *Q*/*WH*, providing a nominal wall shear rate of *γ̇*_w_ = 6*U*/*W*. The reduction in cross-sectional area due to the cylinder causes the flow velocity to increase in the gaps between the cylinder and the channel walls. At the location *x* = 0, the average flow velocity in the gaps is given by *U*_gap_ = *U*/(1 − *B*_R_), and the nominal wall shear rate in the gap is given by *γ̇*_w,gap_ = 6*U*_gap_/0.5*W*(1 − *B*_R_) = 12*U*/*W*(1 − *B*_R_)^2^ = 2*γ̇*_w_/(1 −*B*_R_)^2^.

As mentioned in the Introduction (Section 1), the Weissenberg number can be defined based on either a shear or an extensional deformation rate. Here, consistent with our prior work (*e.g.*, ref. 13, 18), we consider the deformation rate based on the nominal velocity gradient along the channel centreline near the upstream and downstream stagnation points of the cylinder, *i.e.*, the nominal extensional rate in those locations. Hence we consider an extensional Weissenberg number defined as *Wi* = *λU*/*R*. Note that this definition of *Wi* is numerically equivalent to the Deborah number of the flow *De* = *λ*/*T*_flow_, where *T*_flow_ = *R*/*U* is the characteristic time for the flow to pass the cylinder. Based on this definition, at the maximum values of Weissenberg number tested (*Wi*_max_ = 1000), the average flow velocities are *U* ≈ 7 mm s^−1^ (for the lowest *B*_R_ = 0.055) and *U* ≈ 82 mm s^−1^ (for the highest *B*_R_ = 0.63). Corresponding shear rate values for *B*_R_ = 0.055 are *γ̇*_w_ ≈ 106 s^−1^ and *γ̇*_w,gap_ ≈ 240 s^−1^, while those for *B*_R_ = 0.63 are *γ̇*_w_ ≈ 1230 s^−1^ and *γ̇*_w,gap_ ≈ 17970 s^−1^. An alternative definition of the Weissenberg number, based on the wall shear rate in the gap, can be written *Wi*_gap_ = *λγ̇*_w,gap_ = 12*λU*/*W*(1 − *B*_R_)^2^. For each blockage ratio considered, *Wi*_gap_ ∝ *Wi*, however the constant of proportionality depends on *B*_R_. For instance, at the lowest blockage ratio of *B*_R_ = 0.055, we find that *Wi*_gap_ ≈ 0.37*Wi*, while for the highest blockage ratio of *B*_R_ = 0.63, we find that *Wi*_gap_ ≈ 28*Wi*.

As discussed in ref. 18, we consider the most appropriate definition of the Reynolds number (describing the ratio of inertial to viscous forces in the flow), to be *Re*_0_ = *ρUR*/*η*_0_, where *ρ* = 1000 kg m^−3^ is the fluid density. By this definition, based on the zero shear rate viscosity of the fluid, for the largest cylinder at the highest flow rate imposed in our experiments, we obtain the maximum value *Re*_0,max_ ≈ 4 × 10^−4^. If instead we consider a Reynolds number based on the infinite shear rate viscosity, *Re*_∞_ = *ρUR*/*η*_∞_, we obtain *Re*_∞,max_ ≈ 5. However, for all but the largest cylinders at the highest flow rates tested, *Re*_∞_ < 1. Furthermore, the elasticity number (comparing elastic to inertial forces in the flow) can be defined as *El* = *Wi*/*Re*. Even for the case of the largest cylinder (*i.e.*, highest Re), we find *El*_0_ = *Wi*/*Re*_0_ ≈ 2.7 × 10^6^, while *El*_∞_ = *Wi*/*Re*_∞_ ≈ 200. Since the Reynolds number is small and *El*_∞_ ≫ 1, we consider that inertia plays a negligible role in determining the flow dynamics.

### 2.3 Flow velocimetry

Quantitative and spatially-resolved 2D velocity fields are acquired using micro-particle image velocimetry (μ-PIV, TSI Inc.).^26,27^ For these measurements, the fluid is seeded with fluorescent tracer particles (2 μm-diameter, Fluoromax red, Thermo Scientific Inc.) at a low concentration (≈0.02 wt%) that does not affect the fluid rheology. Measurements are made by focussing on the mid-plane of the channel (*z* = 0) using an inverted microscope (Nikon Ti) with a 5× magnification, NA = 0.15 numerical aperture Nikon Plan Fluor objective lens. The fluid volume is illuminated by an Nd:YLF dual-pulsed laser (wavelength 527 nm), which is synchronized with a high speed camera (Phantom Miro) working in frame-straddling mode. With this camera and microscope objective, the field of view is 2.05 × 1.28 mm, and with the 2 μm seeding particles the measurement depth is 124 μm, (≈0.06*H*). At each imposed flow rate, the flow is held constant for at least 60 seconds, (≈40*λ*) before initiating the recording of image pairs for analysis. For time-steady flows, 250 μ-PIV frame pairs are recorded at 25 pairs-per-second. For time-dependent flows, 1500 frame pairs are recorded at 50 pairs-per-second. In both cases, frame pairs are cross-correlated both individually and by an ensemble average processing algorithm, providing two-component velocity vectors **u** = [*u*,*v*], where *u* and *v* are the *x* and *y* components of the velocity, respectively. The cross-correlation is performed using OpenPIV, an open-source Python package.^28^

## 3 Results

### 3.1 Steady flow instabilities around the cylinders

We commence the presentation of our experimental results by describing the flow patterns observed around the cylinders over a lower range of Weissenberg numbers at which the flow remains steady in time. For all blockage ratios examined, for Weissenberg numbers below a first critical value 10 ≲ *Wi*_c1_ ≲ 100 (depending on *B*_R_), the flow field around the cylinder appears approximately symmetric about the *x* and *y* axes. For *Wi* > *Wi*_c1_, the flow destabilizes but continues to remain steady. The nature of this initial instability and the resulting flow patterns also depend on *B*_R_, as will be described in detail in the following.

#### 3.1.1 Low blockage ratio, *B*_R_ ≤ 0.33

**Fig. 2:**
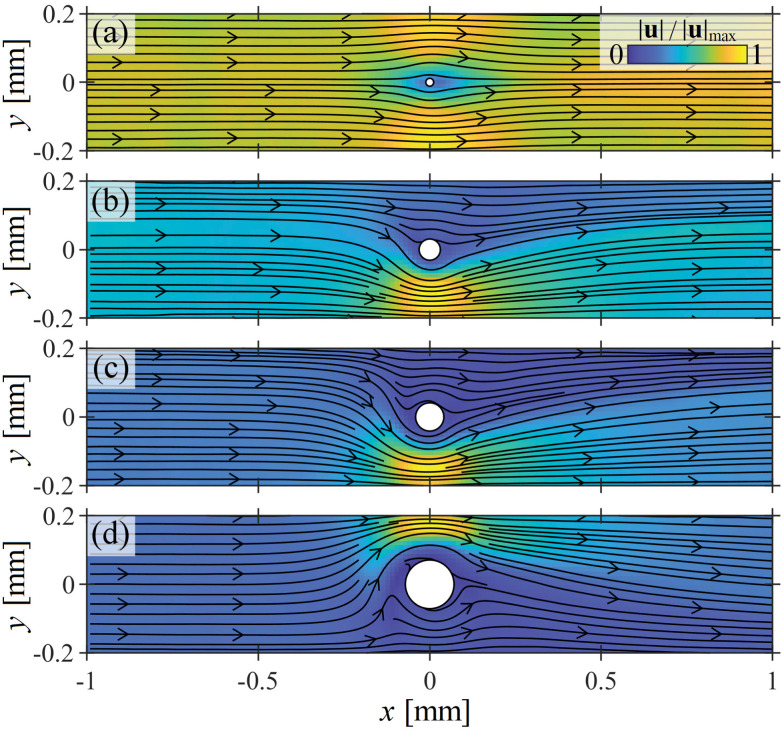
Flow patterns observed for flow of the WLM solution at *Wi* = 40 around cylinders with ‘low’ blockage ratios: (a) *B*_R_ = 0.055, (b) *B*_R_ = 0.15, (c) *B*_R_ = 0.2, (d) *B*_R_ = 0.33.

In Fig. 2 we present normalized velocity magnitude fields obtained at *Wi* = 40 for selected cylinder geometries with a lower range of blockage ratios *B*_R_ ≤ 0.33. For *B*_R_ = 0.055 (Fig. 2(a)), *Wi* = 40 is below the critical value, and the flow field is approximately symmetric. For an increased *B*_R_ = 0.15 (Fig. 2(b)), the imposed Weissenberg number is beyond *Wi*_c1_ and an instability is evident in the form of a strong lateral flow asymmetry. As the blockage ratio is further increased to *B*_R_ = 0.2 (Fig. 2(c)) and *B*_R_ = 0.33 (Fig. 2(d)) the lateral asymmetry increases in intensity. Such asymmetric flow patterns were only quite recently reported for the first time for a WLM solution around a microscale cylinder with *B*_R_ = 0.1, for which *Wi*_c1_ ≈ 60.^13^ Since then, similarly unstable flow configurations have been reported in experiments with polymer solutions,^14,15^ and in numerical simulations employing various constitutive models.^15,29,30^ Currently, it is thought that the asymmetry is initiated by an elastic instability caused by strong elastic stresses near the downstream stagnation point, and is maintained by the resulting imbalance in shear rate (hence viscosity, due to the shear thinning) on either side of the cylinder.^5^ It is important to clarify that for each blockage ratio the preferred flow path occurs randomly on either side of the cylinder.

Fig. 2 shows that by increasing the blockage ratio in the range 0.055 ≤ *B*_R_ ≤ 0.33, the system transitions from a steady symmetric state into a state of increasingly intense lateral asymmetry. For a fixed value of *B*_R_, and increasing the Weissenberg number, the flow also transitions from symmetric to asymmetric (as shown in a prior publication with *B*_R_ = 0.1).^13^ Both *Wi* and *B*_R_ can be used as control parameters to vary the degree of lateral asymmetry.

For each value of *B*_R_ examined, we quantify the degree of flow asymmetry *via* the rate of flow around either side of the cylinder using the following asymmetry parameter:

1 *I* = |*u*_+_ −*u*_−_|/(*u*_+_ + *u*_−_),

where *u*_+_ and *u*_−_ are the integrated values of *u* along the line *x* = 0 for *y* > 0 and *y* < 0, respectively. A value of *I* = 0 indicates that flow is passing the cylinder symmetrically, whereas a value of *I* = 1 indicates that all of the fluid passes the cylinder on either one side or the other.

**Fig. 3:**
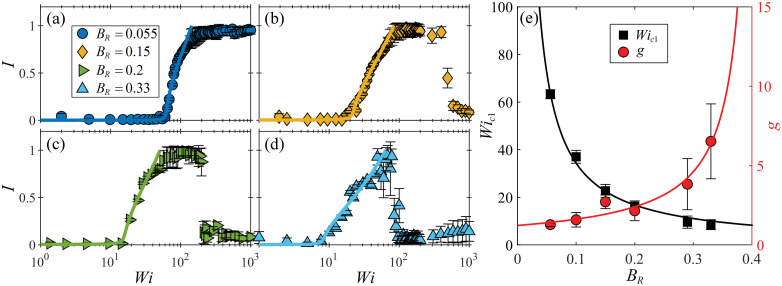
Characterization of the lateral flow asymmetry observed around cylinders presenting low blockage ratios *B*_R_ ≤ 0.33. Asymmetry parameter *I* (eqn (1)) as a function of *Wi* for the same four *B*_R_ cases shown in Fig. 2: (a) *B*_R_ = 0.055, (b) *B*_R_ = 0.15, (c) *B*_R_ = 0.2, (d) *B*_R_ = 0.33. Data points are experimental measurements, solid lines are the best fits of the Landau model (eqn (2)). (e) Fitting parameters extracted from the Landau model for all tested blockage ratios in the range 0.055≤ *B*_R_ ≤0.33. Solid lines are fits to the experimental data points of the form *Wi*_c1_ ∼ 1/*B*_R_ (black), and *g* ∼ 1/(*a* − *B*_R_), where *a* = 0.41 (red), see main text.

Fig. 3(a–d) shows examples of the asymmetry parameter *I* plotted as a function of the imposed Weissenberg number for the same four blockage ratio cases illustrated in Fig. 2. For *B*_R_ = 0.055 (Fig. 3(a)), as the Weissenberg number is increased the flow becomes laterally asymmetric at *Wi*_c1_ ≈ 70, and subsequently stays strongly asymmetric (with *I* ≈ 1) over the remaining range of *Wi* probed (*Wi* up to ≈1000). As *B*_R_ is progressively increased through Fig. 3(b–d), the value of *Wi*_c1_ progressively decreases, and the rate of growth of the asymmetry decreases. In each case, for *Wi* > *Wi*_c1_ the flow becomes strongly asymmetric, but the range of *Wi* over which the asymmetry is maintained is progressively reduced as *B*_R_ increases. At higher *Wi*, the flow transitions back towards a laterally symmetric state (*I* → 0).

Consistent with prior work at *B*_R_ = 0.1,^13^ the transition to asymmetry is well described using a minimized quartic Landau potential:

2 *Wi* = *Wi*_c1_(*gI*^2^ + *hI*^−1^ + 1),

as shown by the solid lines in Fig. 3(a–d). Eqn (2) allows extraction of the critical Weissenberg number *Wi*_c1_, along with the growth rate coefficient of the asymmetry *g* for each blockage ratio. Note that the coefficient *h* for the asymmetric term quantifies system imperfections that could bias the asymmetry to one side of the cylinder or the other. For all of the blockage ratios tested, *h* is small (|*h*|/*g* ≲ 10^−4^), explaining the apparently random selection of preferred flow path.

The values of *Wi*_c1_ and *g* obtained for each value of *B*_R_ are plotted as a function of *B*_R_ in Fig. 3(e). Here, the error bars represent a one standard deviation confidence interval determined from the least-squares curve fitting of eqn (2) to the experimental data. The critical Weissenberg number is well described by *Wi*_c1_ = 3.3/*B*_R_, in agreement with the 1/*B*_R_ scaling predicted by McKinley and coworkers,^31,32^ and also recently shown for this instability by numerical simulations with the linear Phan–Thien–Tanner model.^15^ This is a further experimental confirmation that the instability is induced by the growth of elastic tensile stress along the curved streamlines near the downstream stagnation point.^5^ The instability growth rate parameter is well described by *g* = 0.5/(0.41 − *B*_R_). Interestingly, this suggests that *g* → ∞ as *B*_R_ → 0.41, indicating that the instability should disappear (infinitely slow growth).

#### 3.1.2 High blockage ratio, *B*_R_ ≥ 0.44

**Fig. 4:**
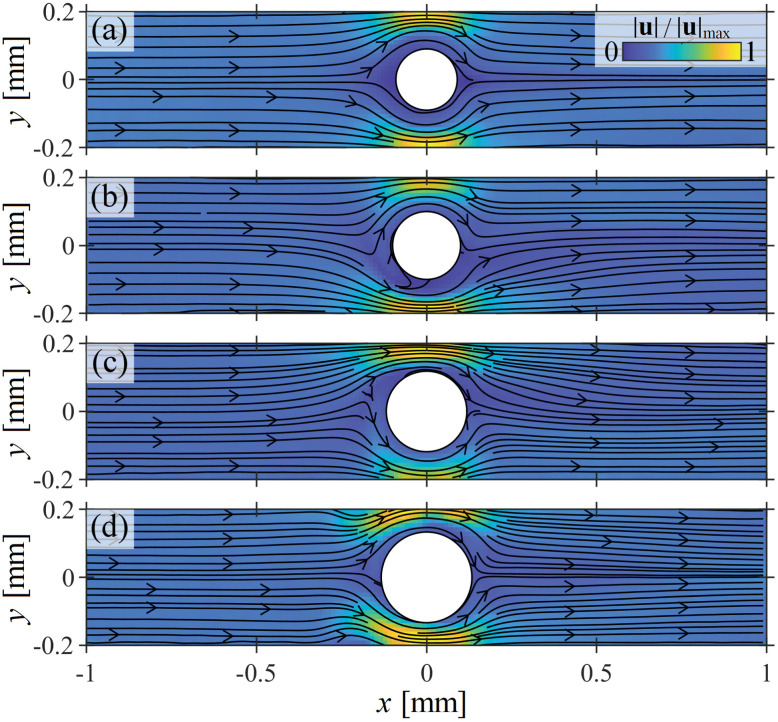
Flow patterns observed for flow of the WLM solution at *Wi* = 30 around cylinders with ‘high’ blockage ratios: (a) *B*_R_ = 0.44, (b) *B*_R_ = 0.48, (c) *B*_R_ = 0.58, (d) *B*_R_ = 0.63.

In Fig. 4 we present velocity magnitude fields obtained for flow of the WLM test fluid at *Wi* = 30 around cylinders presenting a higher range of blockage ratios 0.44 ≤ *B*_R_ ≤ 0.63. This figure illustrates the range of time-steady flow states observed for ‘high’ blockage ratios close to a first instability occurring at *Wi*_c1_. At the lowest blockage ratio shown (*B*_R_ = 0.44, Fig. 4(a)), *Wi* = 30 < *Wi*_c1_ and the flow field is approximately symmetric about *x* = 0 and *y* = 0. For *B*_R_ ≥ 0.48, *Wi* = 30 > *Wi*_c1_ and flow instabilities are manifested, although distinct from those shown in Fig. 2 for *B*_R_ ≤ 0.33. At *B*_R_ = 0.48 (Fig. 4(b)), downwards bending streamlines can be observed upstream of the cylinder, while at *B*_R_ = 0.58 (Fig. 4(c)), upwards bending streamlines are observed. Finally, at the highest blockage ratio shown (*B*_R_ = 0.63, Fig. 4(b)) the flow field appears to have regained symmetry about *y* = 0, but a close inspection reveals small perturbations of the streamlines on the walls upstream of the cylinder. These are where upstream wall-attached vortices are beginning to form, as first reported in a recent work for flow of the same WLM solution past a cylinder with *B*_R_ = 0.5.^18^ As discussed in ref. 18, the formation of upstream wall vortices serves to remove the ‘kink’ in the streamlines near the upstream stagnation point.

**Fig. 5:**
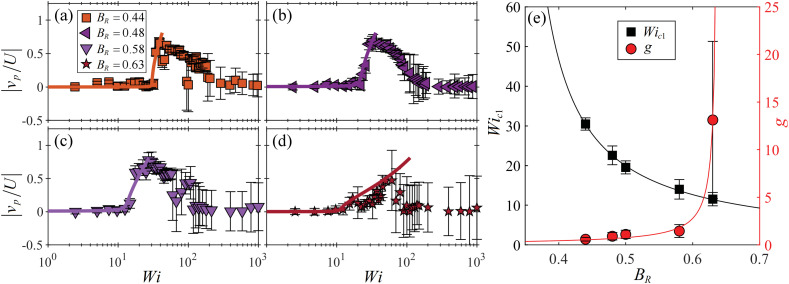
Characterization of the bending streamlines instability observed for flow around cylinders presenting high blockage ratios *B*_R_ ≥ 0.44. Normalized magnitude of the transverse component of the velocity at an upstream probe location |*v*_p_/*U*| (see text for full description) as a function of *Wi* for the same four *B*_R_ cases shown in Fig. 4: (a) *B*_R_ = 0.44, (b) *B*_R_ = 0.48, (c) *B*_R_ = 0.58, (d) *B*_R_ = 0.63. Data points are experimental measurements, solid lines are the best fits of the Landau model (eqn (3)). (e) Fitting parameters extracted from the Landau model for all tested blockage ratios in the range 0.44 ≤ *B*_R_ ≤ 0.63. Solid lines are fits to the experimental data points of the form *Wi*_c1_∼ 1/*B*_R_ (black), and *g* ∼ 1/(*a* − *B*_R_), where *a* = 0.64 (red), see main text.

We quantify the bending streamline instability observed at higher blockage ratios by extracting a probe velocity *v*_p_, which is the *y*-component of the flow velocity at a probe location (*x* = −1.4*R*) upstream of the cylinder. The data points in Fig. 5(a–d), show |*v*_p_/*U*| measured as a function of *Wi* for the same four blockage ratio cases presented in Fig. 4.

The solid lines in Fig. 5(a–d) are fits of the following Landau equation:

3 *Wi* = *Wi*_c1_[*g*(|*v*_p_/*U*|)^2^ + *h*(|*v*_p_/*U*|)^−1^ + 1],

from which *Wi*_c1_ and *g* are extracted. As before for the low *B*_R_ cases, the asymmetric *h* coefficient is again extremely small for each blockage ratio, and the streamlines can bend either upwards or downwards with apparent randomness.

At *B*_R_ = 0.44 (Fig. 5(a)), the onset of streamline bending is extremely abrupt at *Wi*_c1_ ≈ 30, at which |*v*_p_/*U*| increases rapidly to a maximum, before gradually decreasing with further increasing *Wi*. Increasing the blockage through *B*_R_ = 0.48 (Fig. 5(b)), *B*_R_ = 0.58 (Fig. 5(c)), and *B*_R_ = 0.63 (Fig. 5(d)), the transition occurs at progressively lower *Wi*_c1_ and becomes more gradual (*i.e.*, *g* increases).

Fig. 5(e) shows *Wi*_c1_ and *g* plotted as a function of *B*_R_ for all channels tested with *B*_R_ ≥ 0.44. As before for the lower *B*_R_ cases, we find a 1/*B*_R_ dependence for both *Wi*_c1_ and *g* with *Wi*_c1_ = 3.5/(*B*_R_ − 0.33) and *g* = 0.1/(0.64 − *B*_R_). The scaling *Wi*_c1_ ∼ 1/*B*_R_ is again consistent with the prediction of an elastic instability driven by a combination of elastic tensile stress and streamline curvature,^31,32^ in this case the location of importance clearly being the upstream stagnation point. The asymptote in *Wi*_c1_ at *B*_R_ = 0.33 suggests that bending streamlines upstream of the cylinder should not be observed for lower blockage ratios than this. The asymptote in *g* at *B*_R_ = 0.64 further suggests that the bending streamline instability should vanish at even higher blockage ratios. Unfortunately, we were unable to test this due to difficulty in fabricating devices with *B*_R_ > 0.63.

Comparing the asymptotes found for ‘low’ *B*_R_ and ‘high’ *B*_R_ behaviour (Fig. 3(e) and 5(e), respectively) indicates the presence of an ‘intermediate’ blockage ratio regime for 0.33 ≲ *B*_R_ ≲ 0.41, where the lateral asymmetry and the upstream bending streamline instabilities could coexist.

#### 3.1.3 Intermediate blockage ratio, example case, *B*_R_ = 0.4

**Fig. 6:**
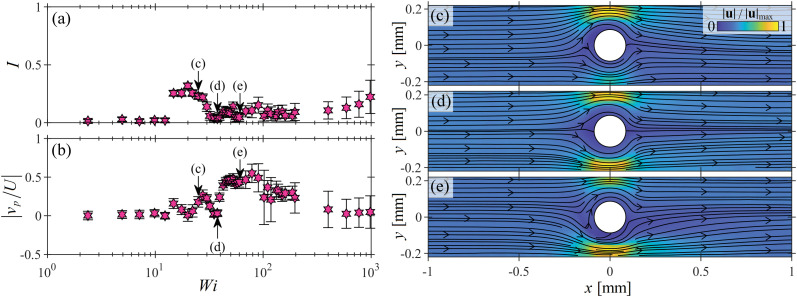
Example of flow behaviour evolution with *Wi* at an ‘intermediate’ blockage ratio of *B*_R_ = 0.4. (a) Asymmetry parameter *I*, and (b) normalized probe velocity |*v*_p_/*U*| as a function of *Wi*. Panels (c–e) show the velocity magnitude fields measured at the correspondingly-labeled points in parts (a and b), with *Wi* = 22, 40, and 60, respectively.

Fig. 6(a and b) show the asymmetry parameter *I* and normalized probe velocity |*v*_p_/*U*| (respectively) *versus Wi* for an example intermediate blockage ratio case of *B*_R_ = 0.4. There is an abrupt jump in *I* at *Wi* ≈ 12, indicating the onset of a laterally asymmetric flow state, as typically observed at ‘low’ *B*_R_. Note that |*v*_p_/*U*| is also nonzero at *Wi* = 12, but its value is relatively small compared to *I*. At a higher *Wi* = 22 (marked (c)), both *I* and |*v*_p_/*U*| have similar nonzero values. The corresponding velocity field shown in Fig. 6(c) shows both a lateral asymmetry reminiscent of ‘low’-*B*_R_ behaviour, and a kinked streamline near the upstream stagnation point reminiscent of ‘high’-*B*_R_ behaviour. Further increasing the Weissenberg number to *Wi* = 40 (marked (d)), both *I* and |*v*_p_/*U*| are close to zero, and the corresponding velocity field shown in Fig. 6(d), appears rather symmetric. At slightly higher *Wi*, |*v*_p_/*U*| increases abruptly while *I* remains low. At *Wi* = 60 (marked (e)), the corresponding velocity field shown in Fig. 6(e) is largely characterized by upstream bending streamlines, as typically observed at ‘high’ *B*_R_.

In general, for this example intermediate *B*_R_ case, we observe that the transition between flow states with increasing *Wi* is more complex than at either low or high *B*_R_. Although we see both flow behaviours that could be classified as ‘lateral asymmetry’ or ‘upstream bending streamlines’, neither progresses with increasing *Wi* in the same way as seen when the blockage ratio is clearly either ‘low’ or ‘high’. For this intermediate *B*_R_ = 0.4 case, there appears to be a competition between the instabilities that arise at low and high *B*_R_.

For low *B*_R_, the lateral flow asymmetry is initiated due to the accumulation of elastic tensile stress on the streamlines that curve near the downstream stagnation point of the cylinder, in accordance with the prediction of McKinley and coworkers.^5,15,32^ For high *B*_R_, it appears that elastic tensile stress on the streamlines curving near the upstream stagnation point drive the onset of instability there, too. Within the intermediate regime it seems that neither stagnation point clearly dominates and that both stagnation points actively contribute to the observed dynamics, competing for dominance in a complex interplay.

## 4 Time-dependent fluctuations

As the Weissenberg number is increased well beyond *Wi*_c1_, the flow eventually exhibits time-dependent features. In this section, we provide a brief discussion of the time-dependence observed above a second critical Weissenberg number *Wi*_c2_ at various blockage ratios. A thorough analysis of the time-dependence is beyond the scope of this work, but two prior works provide detailed studies, including spectral analysis, performed at low *B*_R_ = 0.1,^13^ and high *B*_R_ = 0.5.^18^ Here we mostly seek to find the approximate value of *Wi*_c2_ for each blockage ratio and to broadly categorise the observed flow states. Although the flow likely fluctuates in all three spatial directions,^18^ we assume that this occurs simultaneously in each direction at a coincident Weissenberg number. Therefore we may find *Wi*_c2_ with reasonable accuracy based on our 2D flow velocimetry. To this end, we use time-resolved 2D velocity fields to approximately quantify the turbulence intensity *T* as a function of the imposed *Wi*. The turbulence intensity is defined as:4
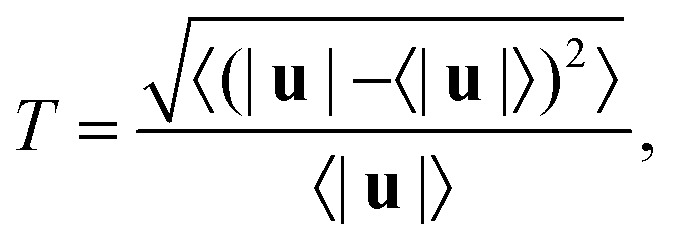
where 〈〉 indicates a time-average.

**Fig. 7:**
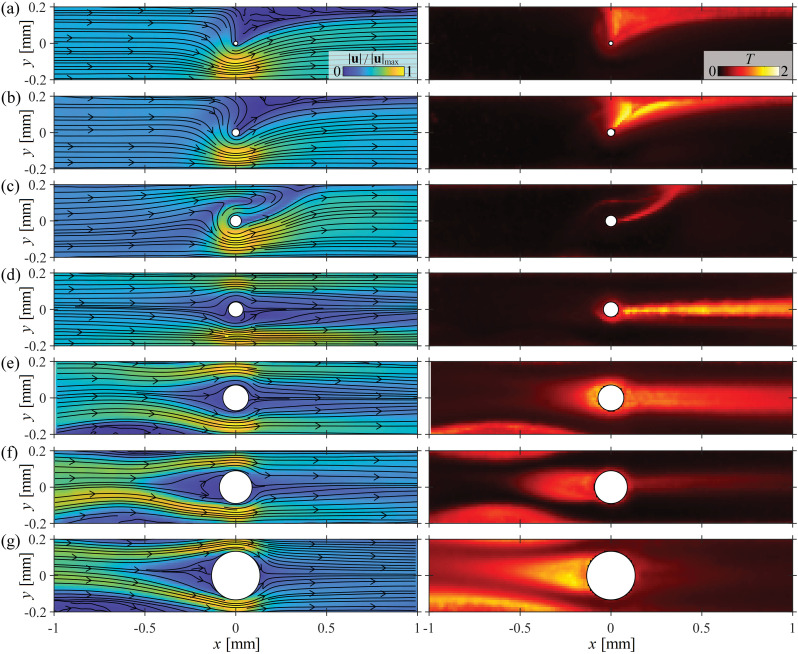
Illustration of time-dependent flow states observed in devices with various blockage ratios. Time-averaged velocity magnitude fields (left side), and corresponding turbulence intensity fields (right side) obtained for time-dependent flows at *Wi* ≈ 400 around cylinders presenting various blockage ratios: (a) *B*_R_ = 0.055, (b) *B*_R_ = 0.1, (c) *B*_R_ = 0.15, (d) *B*_R_ = 0.2, (e) *B*_R_ = 0.33, (f) *B*_R_ = 0.44, (g) *B*_R_ = 0.63.

In Fig. 7, we present time-averaged velocity magnitude fields along with their corresponding turbulence intensity fields obtained at *Wi* ≈ 400 in devices of various blockage ratio. At the three lowest values of *B*_R_ = 0.055, 0.1, and 0.15 (Fig. 7(a)–(c), respectively), we observe a time-dependent lateral asymmetry, where the flow mostly fluctuates within the low-velocity wedge of fluid located at positive *y*. At this *Wi*, with increasing *B*_R_, that fluctuating wedge of fluid becomes compressed as the streamlines push further past the cylinder into the downstream region before doubling back. For *B*_R_ = 0.15 (Fig. 7(c)), the low-velocity region has become completely pinched resulting in a thin layer of sheared fluid that curves from the rear stagnation point of the cylinder towards the top wall of the channel at *y* = 0.2 mm. All of the flow fluctuations occur in this strongly sheared region. Increasing the blockage further to *B*_R_ = 0.2 (or alternatively if *Wi* is increased further at *B*_R_ = 0.15), the flow is able to push past the cylinder on both sides of the channel (although unequally) resulting in ‘asymmetric jets’ of high flow velocity extending downstream near the walls of the channel (Fig. 7(d)). All of the flow fluctuations are concentrated in the wake behind the cylinder where the flow velocity is generally low, but frequent pulses of high velocity occur. Further increasing the blockage ratio to *B*_R_ = 0.33, 0.44, and 0.63 (Fig. 7(e)–(g), respectively), progressively larger wall vortices form upstream of the cylinder and the flow fluctuations in the wake decrease progressively in intensity. A vortex forms at the upstream stagnation point of the cylinder and there are flow fluctuations in both the wall-attached and cylinder-attached vortices. As reported in a prior publication at *B*_R_ = 0.5,^18^ when there is an upstream cylinder-attached vortex, it competes with the wall-attached vortices for time and space in the channel. In some of the present experiments only a cylinder vortex is observed over the entire 20 s duration of the data acquisition. However, in repeated test runs, both cylinder and wall vortices could be observed at various times. In subsequent discussion, we will refer to flow states such as ‘upstream cylinder vortex’ or ‘upstream wall vortex’, however these are not necessarily exclusive. It is possible for multiple flow ‘states’ to be observable for a given pair of *Wi* and *B*_R_.

**Fig. 8:**
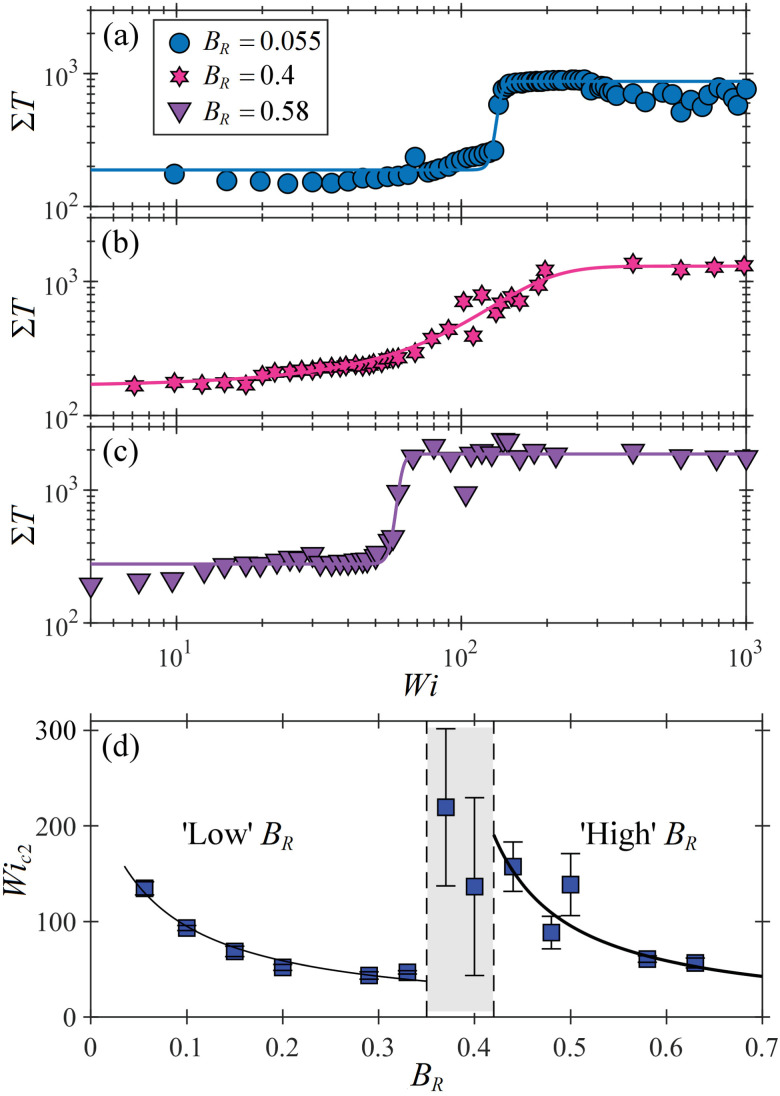
Transition to time dependent flow. Spatially-averaged turbulence intensity *ΣT* as a function of *Wi* for (a) low *B*_R_, (b) intermediate *B*_R_, and (c) high *B*_R_ cylinders. Experimental data (points) are fitted with a hyperbolic tangent function (eqn (5), lines) in order to extract values for *Wi*_c2_ marking the onset of time-dependent flow at each value of *B*_R_. (d) *Wi*_c2_ as a function of *B*_R_ for all blockage ratios tested in the experiments. Data are fitted as *Wi*_c2_ = 15.5/*B*_R_ (low *B*_R_ regime) and *Wi*_c2_ = 15/(0.3 − *B*_R_) (high *B*_R_ regime). The intermediate *B*_R_ regime is shaded gray.

In order to unambiguously determine a value for *Wi*_c2_, at which a flow state becomes time dependent, we spatially average the turbulence intensity over the field of view to obtain a quantity *ΣT*. Fig. 8 shows plots of *ΣT* as a function of *Wi* for three blockage ratios: *B*_R_ = 0.055 (Fig. 8(a), illustrating low *B*_R_ behaviour), *B*_R_ = 0.4 (Fig. 8(b), illustrating intermediate *B*_R_ behaviour), and *B*_R_ = 0.58 (Fig. 8(c), illustrating high *B*_R_ behaviour). For each blockage ratio there is a clear increase in *ΣT* as the flow becomes time dependent. For the low and high *B*_R_ cases, the increase is step-like, but at intermediate *B*_R_ the increase is more gradual. The experimental data is fitted with a hyperbolic tangent function given by:5
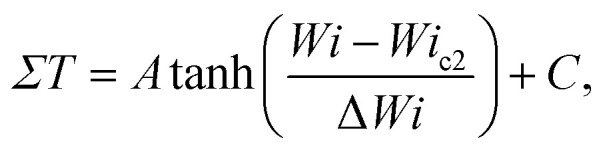
where Δ*Wi* controls the ‘width’ of the transition, *Wi*_c2_ marks the midpoint of the increase in *ΣT*, and *A* and *C* are additional fitting constants. Determining *Wi*_c2_ by this method for all blockage ratios tested, we obtain the plot of *Wi*_c2_ as a function of *B*_R_ shown in Fig. 8(d), where the error bars represent Δ*Wi*. In the low and high *B*_R_ regimes, the data again show a 1/*B*_R_ dependence (though with some scatter in the data at high *B*_R_). Clear outliers that do not fit the trends found at either low or high *B*_R_ are evident in the intermediate regime (shaded gray region), where the error bars are large because of the broad transition to time dependence. We suggest that for low *B*_R_ the dominance of the downstream stagnation point enables elastic stresses to grow sufficiently to trigger an abrupt Hopf bifurcation to a time dependent flow state that pulses with a period similar to the terminal relaxation time of the wormlike micelles.^13^ For high *B*_R_, a similar argument can be made for elastic stress near the upstream stagnation point triggering a transition to a time-dependent state with a period commensurate with the breakage timescale of the micelles.^18^ However, at intermediate *B*_R_, the two stagnation points compete for influence in the flow, ultimately limiting significant growth of elastic stress at either location and rendering the transition to time dependence smooth. Note that for both low *B*_R_^13^ and for high *B*_R_,^18^ higher harmonics appear in the power spectra of the fluctuations as the Weissenberg number is increased beyond *Wi*_c2_. In neither case does the flow exhibit the expected power spectra characteristic of the chaotic flow state known as “elastic turbulence”,^33,34^ even for very high *Wi*. At low *B*_R_, the fluctuations for *Wi* ≫ *Wi*_c2_ exhibit a single characteristic frequency similar to the micelle breakage rate.^13^ For high *B*_R_, a power-law decay is observed in the power spectrum, suggesting chaotic dynamics, but with a slope too shallow to indicate elastic turbulence.^18^ It is likely that micelle breakage at high *Wi* limits the growth of elastic stresses,^35^ thus suppressing the onset of elastic turbulence in micellar systems.

## 5 Flow state diagram

**Fig. 9:**
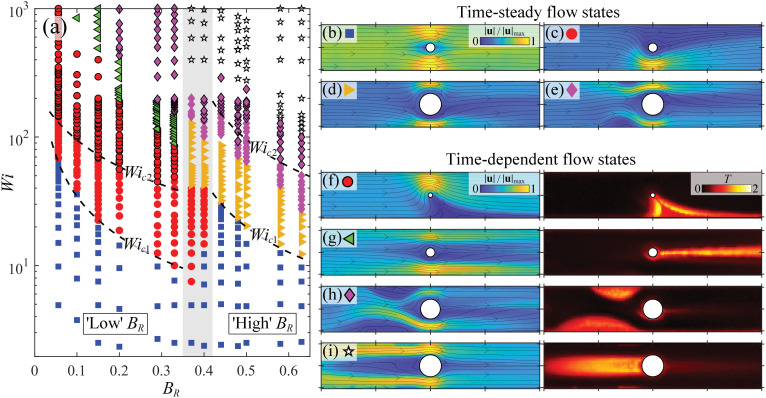
(a) Flow state diagram in *Wi*–*B*_R_ state space. Coloured symbols represent different steady and time-dependent flow states depicted to the right, as indicated by corresponding symbols: (b) low-*Wi* ‘symmetric’ state (*B*_R_ = 0.2, *Wi* = 9.5); (c) ‘laterally asymmetric’ state (*B*_R_ = 0.2, *Wi* = 43); (d) ‘upstream bending streamlines’ (*B*_R_ = 0.48, *Wi* = 53); (e) ‘upstream wall vortices’ (*B*_R_ = 0.48, *Wi* = 82); (f) ‘time-dependent laterally asymmetric’ state (*B*_R_ = 0.1, *Wi* = 257); (g) ‘asymmetric jetting’ (*B*_R_ = 0.2, *Wi* = 384); (h) ‘time-dependent upstream wall vortices’ (*B*_R_ = 0.44, *Wi* = 160); (i) ‘upstream cylinder vortex’ (*B*_R_ = 0.48, *Wi* = 481).

Finally, we summarise our experimental results by constructing a flow state diagram in *Wi*–*B*_R_ parameter space, as shown in Fig. 9(a). Here the different symbols refer to different observed steady and time-dependent flow states illustrated alongside in Fig. 9(b–i), and identified by corresponding symbols. The stability boundaries marked by dashed lines at *Wi*_c1_ and *Wi*_c2_ are the fits to the experimental data shown in Fig. 3(e), 5(e) and 8(d). The intermediate *B*_R_ regime is shaded gray in Fig. 9(a), and marks a striking change in behaviour between the low and high *B*_R_ regimes. For instance, increasing *B*_R_ across the intermediate range at a fixed Weissenberg number (say *Wi* = 20), the flow state switches from ‘laterally asymmetric’ (Fig. 9(c)) to ‘symmetric’ (Fig. 9(b)). Similarly, at a higher Weissenberg number (say *Wi* = 100), increasing *B*_R_ across the intermediate range causes the flow to switch from a ‘time-dependent laterally asymmetric’ state (Fig. 9(f)) into a time-steady state characterized by ‘upstream bending streamlines’ (Fig. 9(d)). Note that for increasing *Wi* at a fixed *B*_R_, changes in the flow state, *e.g.*, from ‘time-dependent upstream wall vortices’ (Fig. 9(h)) to ‘upstream cylinder vortex’ (Fig. 9(i)) are not necessarily abrupt or exclusive. For instance, as discussed above, an upstream cylinder vortex can appear at a given *Wi* and coexist with upstream wall vortices over a certain range of *Wi*. Similarly, the ‘asymmetric jetting’ state Fig. 9(g) may coexist with upstream wall vortices. The flow state indicated by a given symbol in Fig. 9(a) represents the ultimate state to be observed as *Wi* is increased to the given value at the given *B*_R_. For the interested reader, the ESI† includes an alternative flow state diagram presented in *Wi*_gap_–*B*_R_ parameter space (Fig. S1, ESI†), where *Wi*_gap_ is computed based on the shear rate between the cylinder and the channel wall (as described in Section 2.2). While, compared to Fig. 9(a), this results in a *B*_R_-dependent shift in the boundaries between flow transitions, the low and high *B*_R_ regimes remain clearly demarcated.

## 6 Discussion and conclusions

We have presented microfluidic experiments on the flow of a viscoelastic wormlike micellar solution around cylinders presenting a wide range of blockage ratios 0.055 ≤ *B*_R_ ≤ 0.63. The experiments have been performed at negligible Reynolds numbers (*i.e.*, vanishing inertia) and over a wide range of the Weissenberg number (*Wi* up to 1000). Experiments on flows around cylinders in channels (a benchmark geometry for studying non-Newtonian flows) have never before been carried out over such a wide range of *Wi*–*B*_R_ parameter space. Our experiments reveal a rich variety of steady and time-dependent flow states that can be observed depending on both *Wi* and *B*_R_. For *B*_R_ ≲ 0.33 (*i.e.*, ‘low’ *B*_R_), flow instabilities originate from the downstream stagnation point. An initial instability at *Wi*_c1_ ∼ 1/*B*_R_ marks a transition from a symmetric to a laterally asymmetric, but time-steady flow state. Above a higher critical value *Wi*_c2_ (also ∼1/*B*_R_), the flow becomes time dependent, exhibiting strong fluctuations downstream of the cylinder. For *B*_R_ ≳ 0.44 (*i.e.*, ‘high’ *B*_R_), the observed steady flow instabilities above *Wi*_c1_ ∼ 1/*B*_R_ originate from the upstream stagnation point, initially manifesting as bending streamlines, and subsequently developing into wall-attached vortices upstream of the cylinder. Above *Wi*_c2_, time-dependence in the flow around the high-*B*_R_ cylinders manifests as strong fluctuations in the upstream wall-attached vortices and subsequently in an upstream cylinder-attached vortex that forms as *Wi* is further increased.

It has long been recognised that changing the blockage ratio of the cylinder in the channel can shift the influence of the different features in the flow field, leading to different dynamical flow phenomena.^2,3^ By varying *B*_R_ incrementally over a wide range, we have been able to clearly distinguish the ranges of *B*_R_ that can be considered to be in either the ‘low’ or the ‘high’ *B*_R_ regimes. We also identify a narrow ‘intermediate’ *B*_R_ regime spanning 0.33 ≲ *B*_R_ ≲ 0.41, across which there is a clear shift in behaviour. Within the intermediate *B*_R_ regime, the flow transitions are more complex and less well defined than in either the low- or the high-*B*_R_ regime. This is presumably due to the competing influence between instabilities arising from each stagnation point. In future work, it will be interesting to perform a more detailed study focussing specifically within this intermediate range of *B*_R_ in order to understand this complex behaviour more thoroughly. We suspect there may be some analogies to be made to phase transitions in thermodynamic^36^ or active matter^37^ systems. The distinct boundaries between flow states delineated by the *Wi*_c1_ curves within the low and high *B*_R_ regimes may be similar to spinodal curves. The apparent observation of competing, or merging, flow states within the intermediate *B*_R_ regime may indicate a coexistence regime (analogous to the ‘miscibility gap’) and hence suggests the possible presence of a binodal curve in this region of the flow state diagram. As such, the observed instabilities in the intermediate *B*_R_ regime may be prone to hysteresis or ‘priming’-like behaviour, where the system can become stuck in a given state while the control parameter is varied. Similar behaviour has been observed for the flow of wormlike micellar solutions past side-by-side microcylinders.^38^ Since it is practically impossible to hold *Wi* constant while varying *B*_R_ in an experiment, a numerical study of this system with dynamically varying *Wi* and/or *B*_R_ (such as that performed for viscoelastic flow past low *B*_R_ cylinders *i.e.*, ref. 15) may prove invaluable for the calculation of theoretical boundaries in the phase diagram. Note that the high aspect ratio of our experimental devices, *A*_R_ = 5 greatly facilitates a comparable numerical study since the flow can be reasonably approximated as being two-dimensional.

The present work has revealed that viscoelastic flow in a simple geometry comprised of a single obstacle in a channel is rich in time-steady and time-dependent flow behavior. Like high-Reynolds number inertial flows in geometrically simple systems such as between co- or counter-rotating cylinders,^39^ pipe flow,^40^ and flow past a cylinder,^41^ geometric simplicity does not preclude rich and dynamic flow behaviour as the geometric and flow conditions are varied. The study of inertia-less viscoelastic flow instabilities in microfluidic geometries is growing rapidly with the recent advancement in microfluidic channel fabrication^25^ and promises to yield similarly rich flow behaviour. We believe that the flow state diagram we have constructed can serve as a foundation for future studies on viscoelastic flow past obstacles in microchannels, and aid in the design of microfluidic systems where viscoelastic fluids will be used. In recent prior works on flows past cylinders (*e.g.*, ref. 13–15, 38), we have observed broadly analogous behaviour for both wormlike micellar solutions and for shear thinning viscoelastic polymer solutions (especially in the time-steady regime). However, it would be interesting to employ polymeric fluids to examine how the flow state diagram becomes modified as the degrees of shear thinning and elasticity are fine-tuned by variation of *e.g.*, the polymer concentration or molecular weight, or the solvent quality or viscosity.

## Conflicts of interest

There are no conflicts to declare.

## Supplementary Material

SM-018-D2SM01162J-s001
