# Supplementary material for: Effect of blockage ratio on flow of a viscoelastic wormlike micellar solution past a cylinder in a microchannel
Source: Soft Matter. 2022 Nov 14;18(46):8856–66. doi: 10.1039/d2sm01162j (PMC9709659; doi:10.1039/d2sm01162j)
Supplement: SM-018-D2SM01162J-s001 [file SM-018-D2SM01162J-s001.pdf]

Electronic Supplementary Material (ESI) for Soft Matter.
This journal is © The Royal Society of Chemistry 2022

ELECTRONIC SUPPLEMENTARY INFORMATION

Effect of blockage ratio on flow of a viscoelastic wormlike micellar solution past a cylinder in a microchannel

Cameron C. Hopkins, Amy Q. Shen, and Simon J. Haward

Okinawa Institute of Science and Technology Graduate University, Onna-son, Okinawa 904-0495, Japan

E-mail: c.c.hopkins91@gmail.com, simon.haward@oist.jp

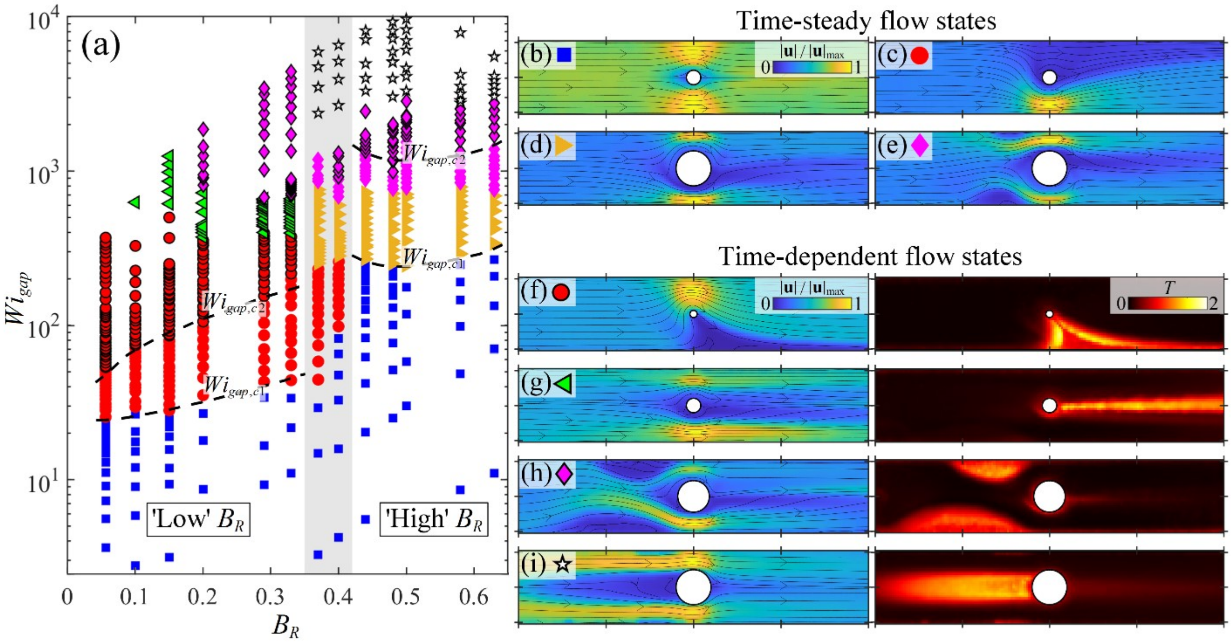

Fig. S1 (a) Flow state diagram in Wi_{gap}-B_R state space. Coloured symbols represent different steady and time-dependent flow states depicted to the right, as indicated by corresponding symbols: (b) low- Wi_{gap} 'symmetric' state (B_R = 0.2, Wi_{gap} = 18); (c) 'laterally asymmetric' state (B_R = 0.2, Wi_{gap} = 81); (d) 'upstream bending streamlines' (B_R = 0.48, Wi_{gap} = 564); (e) 'upstream wall vortices' (B_R = 0.48, Wi_{gap} = 873); (f) 'time-dependent laterally asymmetric' state (B_R = 0.1, Wi_{gap} = 190); (g) 'asymmetric jetting' (B_R = 0.2, Wi_{gap} = 722); (h) 'time-dependent upstream wall vortices' (B_R = 0.44, Wi_{gap} = 1347); (i) 'upstream cylinder vortex' (B_R = 0.48, Wi_{gap} = 5123).
